# Biotic and Abiotic Factors on Rhizosphere Microorganisms in Grassland Ecosystems

**DOI:** 10.3390/microorganisms13122645

**Published:** 2025-11-21

**Authors:** Bademu Qiqige, Yuzhen Liu, Yu Tian, Li Liu, Weiwei Guo, Ping Wang, Dayou Zhou, Hui Wen, Qiuying Zhi, Yuxuan Wu, Xiaosheng Hu, Ming Li, Junsheng Li

**Affiliations:** 1 China Geological Survey Comprehensive Survey Command Center for Natural Resources, Beijing 100055, China; bdmqqg@126.com (B.Q.); ariel_ty@outlook.com (Y.T.); iuli@mail.cgs.gov.cn (L.L.); wangping@bjfu.edu.cn (P.W.); dayouzhou@163.com (D.Z.); herbert_wen@sina.com (H.W.); et_et@126.com (X.H.); lm18910077797@163.com (M.L.); 2 Academy of Animal Science and Veterinary Medicine, Xining 810016, China; liuyzqhu@163.com; 3 Chinese Research Academy of Environmental Sciences, Beijing 100012, China; 220220932760@lzu.edu.cn (Q.Z.); wuyuxuan221@mails.ucas.ac.cn (Y.W.)

**Keywords:** grassland system, rhizosphere microorganisms, abiotic, biotic

## Abstract

Rhizosphere microbiota, serving as pivotal drivers of multifunctionality in grassland ecosystems, are jointly shaped by the dual influences of biotic and abiotic factors. Among biotic components, plant functional types selectively modulate microbial communities through root exudate specificity, while soil fauna (e.g., nematodes and earthworms) drive microbial interaction networks via biophysical disturbances and trophic cascades. However, excessive nematode grazing suppresses the hyphal extension of arbuscular mycorrhizal fungi (AMF). Moderate grazing facilitates the proliferation of ammonia-oxidizing bacteria through fecal input, whereas intensive grazing induces topsoil compaction, leading to a dramatic 40–60% reduction in lipopolysaccharide content in Gram-negative bacteria. Long-term chemical fertilization significantly decreases the fungal-to-bacterial ratio, while organic amendments enhance microbial carbon use efficiency by activating extracellular enzymatic activities. Regarding abiotic factors, the stoichiometric characteristics of soil carbon, nitrogen, and phosphorus directly regulate microbial metabolic strategies. Hydrological dynamics influence microbial respiratory pathways through oxygen partial pressure shifts—drought stress inhibits mycelial network development. Future research should focus on predicting the emissions of gases such as N_2_O (ozone monomer) and optimizing nitrogen fertilizer management to significantly reduce greenhouse gas emissions at the source. The soil organic carbon storage in grassland ecosystems is extremely large. Effective prediction and management can make these soils become important carbon “sinks”, offsetting the carbon dioxide in the atmosphere. At the same time, transcriptomics and metabolic flux analysis should be combined with multi-omics technologies and in situ labeling methods to provide theoretical basis and technical support for developing mechanism-based and predictable grassland restoration and adaptive management strategies from both macroscopic and microscopic perspectives.

## 1. Introduction

In 1904, Lorenz Hiltner pioneered the concept of the rhizosphere [1]—a confined soil zone modulated by plant roots and shaped by intricate interactions among root systems, soil physicochemical properties, and microorganisms. This microdomain functions as both a unique interface for plant–soil-microbe tripartite synergies and a pivotal ecological niche for nutrient uptake and metabolic processes in plants [2]. Despite its spatially restricted dimensions, the rhizosphere is acknowledged as one of Earth’s most sophisticated ecosystems due to its extraordinary functional complexity. Rhizosphere microorganisms, tightly colonizing root surfaces and adjacent soils, develop distinct community structures under microenvironment-specific selective pressures, characterized by taxonomic composition, diversity profiles, and metabolic functionalities that starkly contrast with bulk soil microbiota [3]. Their metabolic activities and byproducts directly or indirectly regulate nutrient acquisition efficiency and developmental trajectories in plants, forming an interdependent rhizosphere-microbe-plant tripartite network. The rhizosphere system is acutely responsive to environmental perturbations: subtle ecological shifts (e.g., elevated CO_2_ levels drive a transition toward r-strategist dominance, Eutrophic microorganisms can grow and reproduce rapidly in resource-rich environments), enriching Acidobacteria, Proteobacteria, and Ascomycota; warming promotes K-strategist (Oligotrophic microorganisms can survive and reproduce in environments with relatively scarce resources) proliferation, marked by increased relative abundances of Basidiomycota and Actinobacteria [4] dynamically reshape microbial diversity patterns and functional potential. Soil organic carbon (SOC), serving as a microbial energy substrate, further modulates the diversity gradients and stability of rhizosphere communities by regulating the abundance and activity of carbon-cycling functional taxa (e.g., carboxydotrophic microbes) [5]. Key environmental drivers—temperature, moisture, and soil C:N ratios—collectively orchestrate the structural assembly, metabolic activity, and ecological functional divergence of rhizosphere microbiomes through differential regulatory mechanisms.

Grassland ecosystems, encompassing approximately 41% of the Earth’s terrestrial surface, represent a critical component of global biomes, particularly due to their central role in soil nutrient cycling, transformation, and regulation [6]. These ecosystems function as integrated entities driven by aboveground-belowground interactions that orchestrate energy flow and biogeochemical cycling. The rhizosphere—a specialized microdomain characterized by dynamic tripartite interactions among plants, soil, and microorganisms—exhibits accelerated material flux and metabolic turnover compared to bulk soil, serving as both a global “hotspot” for nutrient cycling and a pivotal microenvironment for plant nutrient acquisition and metabolism [7]. However, the assembly dynamics and functional outputs of rhizosphere microbial communities are synergistically or antagonistically modulated by biotic factors (e.g., plant-microbe interactions, soil faunal activity) and abiotic drivers (e.g., climatic variability, soil physicochemical gradients) [8]. Therefore, the multi-factor coupling in the rhizosphere region mainly manifests in the interactions among environmental drivers, plant regulation, and microbial responses. Process models (such as CENTURY) can simulate how climate change (such as warming) or human intervention (such as nitrogen addition) alters the soil environment and thereby affects microbial activities and functions [9]. Network models can reveal how these environmental changes reshape the interaction patterns and network structure of microbial communities [10]; Coupling plants with microorganisms: Process models and statistical models (such as structural equation models) can quantify the key pathway by which plants regulate rhizosphere microorganisms through root exudates. The emerging multi-ecosystem microbial community research further reveals how different parts of plants (within the root, in the rhizosphere, and at the leaf interface) “recruit” and coordinate different functional microorganisms to jointly promote plant growth [11]. Recent advancements in high-resolution sequencing technologies (e.g., metagenomics, transcriptomics) and in situ metabolic imaging tools have enabled unprecedented insights into microbial adaptation mechanisms under multifactorial environmental stress, thereby revitalizing assessments of their potential contributions to sustainable grassland management. This synthesis examines the multidimensional interplay of biotic and abiotic drivers, unraveling their collective influence on microbial diversity, network robustness, and ecosystem functionality.

## 2. Influence of Abiotic Factors on Rhizosphere Microorganisms

### 2.1. Effect of Temperature on Rhizosphere Microorganisms

Current global temperatures are increasing at a rate of 0.2 °C per decade [12]. Corresponding alterations in global climatic regimes intensify the hydrological cycle and influence soil temperature and moisture. Soil, intricately linked to Earth’s biogeochemical and hydrological processes, undergoes modified characteristics under even minor climatic shifts, with precipitation and temperature acting as key drivers of soil nutrient dynamics and microbial biogeochemical cycles [13]. Rising temperatures induce shifts in soil physicochemical properties, such as decreased total C, N, and P; dissolved organic C and P; and microbial C, N, and P, thereby impairing microbial diversity and functionality. Notably, rhizosphere and bulk soil microbial communities exhibit divergent responses to warming. Long-term warming experiments demonstrate significant reductions in the diversity and biomass of soil bacteria, fungi, and protozoa under elevated temperatures [14]. Critically, microbial diversity loss under warming is primarily mediated by soil moisture depletion, suggesting exacerbated biodiversity declines under global warming.

Of particular interest is the taxon-specific nature of these effects: spore-forming bacteria exhibit growth advantages under warming conditions. These observations imply that mechanisms driving microbial diversity loss may involve adaptive traits, intensified species competition, and environmental selection pressures. Furthermore, a clear positive correlation exists between microbial diversity and ecosystem functioning, indicating that warming-induced microbial shifts disrupt critical ecological processes. A four-year open-field warming experiment (2018–2021) at Hulun Lake National Grassland Ecosystem Research Station revealed substantial declines in root exudate carbon (19%) and nitrogen (12%) concentrations under warming, alongside compositional changes marked by reduced alcohols, coenzymes, vitamins, phenylpropanoids, and polyketides. These alterations in exudation rates and profiles triggered significant shifts in rhizosphere microbial diversity and community structure, ultimately diminishing the network complexity of bacterial and fungal consortia. Crucially, reduced exudate-derived C/N input and microbial community restructuring downregulated functional genes linked to soil C/N cycling in the rhizosphere, whereas bulk soil exhibited no significant warming effects on soluble organic carbon or microbial functional gene abundance [9]. Complementing these findings, metatranscriptomic analyses of Icelandic natural grassland soils under simulated warming revealed declines in microbial biomass, total C/N/P, and organic C/P alongside shifts in microbial metabolism [15]. Elevated temperatures upregulated metabolic pathways and cell replication activities but downregulated ribosomal biosynthesis, reflecting resource reallocation strategies to sustain high metabolic activity and cell division rates under thermal stress. Such reconfigurations highlight microbial adaptive trade-offs under warming scenarios, with cascading implications for soil biogeochemistry and ecosystem resilience.

### 2.2. The Influence of Moisture on Rhizosphere Microbes

The heterogeneous alteration of precipitation patterns constitutes a critical environmental challenge under global climate change, imposing profound impacts on global biogeochemical cycles [16]. Precipitation variability, particularly drought, rapidly depletes soil moisture content, adversely affecting root growth, nutrient acquisition in plants, and disrupting microbial life activities. Under drought stress, the drastic reduction in soil moisture subjects rhizosphere microbial communities to survival challenges [17]. Drought alters the composition and quantity of root exudates, significantly reshaping rhizosphere microbial dynamics. While drought generally reduces microbial abundance [18], certain studies report stable or even elevated bacterial abundance under drought, with such variations modulated by soil properties [19]. Notably, microbial phylogenetic diversity decreased by 40% in soils experiencing drought for the first time, whereas no significant diversity loss occurred in pre-acclimated soils under recurrent drought. These discrepancies likely stem from differences in drought duration, intensity, and interactive effects of temperature, soil texture, and nutrient availability [20].

Microbial abundance shows divergent responses to drought, its influence on community composition exhibits higher consistency. A robust body of evidence indicates that drought increases the Gram-positive to Gram-negative bacterial ratio [21], with Actinobacteria and Firmicutes demonstrating rhizosphere-specific enrichment compared to bulk soil. Such microbial shifts may be driven by drought-induced changes in root exudate profiles. For instance, host-derived glycerol-3-phosphate shows significant correlations with Actinobacterial glycerol-3-phosphate transporters, suggesting plant-exudate-mediated microbial restructuring during drought adaptation [22]. Concurrently, water limitation constrains microbial activity and metabolism. Under drought, most microbes enter dormancy or reduce metabolic rates to conserve water and energy, thereby diminishing their functional performance. Specifically, drought impairs microbial carbon metabolism and energy acquisition: limited water availability hinders carbon source accessibility, while increased soil organic matter concentration alters carbon utilization efficiency [23]. Certain microbes resort to secondary metabolites as alternative carbon sources, albeit at reduced energy efficiency due to these compounds’ antioxidant properties and lower nutritional quality under stress [24]. Moreover, drought disrupts nitrogen (N) and phosphorus (P) cycling—key aspects of microbial functional metabolism. Water deficit inhibits organic N and P mineralization, reducing plant-available inorganic N and P release, which constrains plant growth [18]. Drought also skews redox equilibria in N and P transformations, further compromising microbial cycling efficiency. Microbial decomposition capacity is typically suppressed under drought due to metabolic constraints, coupled with reduced rhizodeposition and accumulating undecomposed organic matter, ultimately impairing nutrient cycling sustainability [25]. Therefore, drought has a long-term negative impact on the microorganisms in the rhizosphere of plants, restricting their growth and further hindering them from performing their functions

### 2.3. The Influence of Soil Nutrient on Rhizosphere Microbes

#### 2.3.1. The Influence of Soil Carbon on Rhizosphere Microbes

Globally, over 10% of atmospheric CO_2_ is transferred to soils via plant root deposition annually, exceeding current fossil fuel carbon emissions by an order of magnitude [26]. Consequently, root-derived inputs—including exudates (free sugars, amino acids, and organic acids), mucilage, and sloughed cells—play a pivotal role in soil organic carbon (SOC) sequestration. However, the linkage between root-deposited carbon and SOC remains one of the least understood components in terrestrial carbon cycling [27]. The mechanistic basis for how root-deposited carbon becomes stabilized as SOC during plant growth is still poorly characterized. Rhizosphere processes represent a critical pathway through which plants influence terrestrial ecosystem carbon cycling. As the primary habitat for microbial growth, reproduction, and metabolism, rhizosphere soil is intrinsically linked to biogeochemical cycles and serves as a nexus for maintaining ecosystem processes such as elemental nutrient cycling [28]. The rhizosphere carbon cycle, functioning as a key ecosystem process, modulates SOC sequestration while exhibiting feedback to climate change and atmospheric CO_2_ levels, given that root-associated microbial activity accounts for up to 50% of global ecosystem CO_2_ efflux [29]. Global meta-analyses demonstrate that rhizosphere effects positively influence SOC and microbial biomass carbon: rhizosphere soils exhibit higher SOC content than bulk soils due to intensified root exudation and necromass deposition, yet concurrently display elevated CO_2_ efflux and respiration rates driven by enhanced enzyme activity and nutrient availability [30]. Labile organic carbon fractions (e.g., microbial-derived and soluble organic carbon) readily fuel rhizosphere microbial activity, while root-exuded compounds (amino acids, sugars, organic acids) provide critical energy substrates. Thus, root deposition and photosynthetic carbon allocation belowground jointly regulate SOC stocks through rhizosphere dynamics. The rhizosphere microorganisms are highly “activated” by root exudates (such as sugars and amino acids), and their metabolic activity increases sharply. These microorganisms not only decompose the exudates themselves but may also secrete more enzymes to break down soil organic matter that was previously difficult to decompose and stable, thereby releasing ancient soil carbon in the form of CO_2_ [31]. The same microorganisms activated by root exudates will assimilate the carbon and nitrogen secreted by plants into their own biomass (cell structure). When these microorganisms die, their residues (especially cell walls and viscous extracellular polymers) can be adsorbed and encapsulated by soil mineral particles (such as clay, metal oxides), forming mineral-bound organic matter, which can then be transformed into very stable soil organic carbon and remain in the soil for a long time [32].

Notably, root deposition may trigger the rhizosphere priming effect (RPE) through short-term alterations in native SOC decomposition rates following additional carbon inputs. This priming effect—whether accelerating or inhibiting soil organic matter decomposition—is governed by root exudate-mediated microbial turnover. Specific microorganisms utilize root-derived compounds as metabolic substrates, stimulating the activity of other microbial groups to decompose native SOC [33]. Furthermore, root water uptake intensifies soil drying-rewetting cycles, potentially disrupting existing aggregates and forming new ones. Plant nitrogen uptake increases microbial N demand, driving microbes to exploit N-rich SOM as a compensatory strategy, thereby destabilizing SOC [34]. These mechanisms reveal a critical paradox: rhizosphere interactions can exert dual effects on SOC, either enhancing stabilization through microbial processing or promoting destabilization via priming and nutrient competition.

#### 2.3.2. The Influence of Soil Nitrogen on Rhizosphere Microbes

Nitrogen (N) is a critical growth-limiting nutrient for plants in global terrestrial ecosystems [35]. A key mechanism through which vegetation influences soil organic carbon (SOC) decomposition is the allocation of photosynthate-derived carbon to soils via root exudation [36]. Rhizodeposits are well-documented to accelerate SOC mineralization by stimulating extracellular enzyme production and disrupting organo-mineral associations—a phenomenon termed the rhizosphere priming effect (RPE), which correlates with enhanced plant N uptake efficiency, elevated soil N mineralization rates, and accelerated microbial biomass turnover [37]. Consequently, accounting for rhizospheric processes is essential for elucidating soil N dynamics and plant N acquisition strategies. Greater rhizodeposition intensifies soil N cycling via SOC decomposition, linked to faster total N mineralization and microbial N turnover. This rhizosphere-accelerated N cycling enables plants to assimilate more soil N through roots, which is subsequently allocated to aboveground tissues to support higher photosynthetic carbon assimilation, thereby sustaining accelerated plant growth. Elevated plant N uptake further stimulates microbial activity, reinforcing the rhizosphere priming effect and triggering microbial acquisition of N from SOC pools [38]. The “microbial N mining” hypothesis posits that under N-limiting conditions, rhizosphere microbes utilize labile root-derived C to produce extracellular enzymes that catalyze SOC decomposition, accessing N embedded within organic matter [33]. This process reduces microbial biomass N and C pools while accelerating their turnover. Notably, some plant species prioritize extracellular enzyme production even at the expense of microbial symbionts, exhibiting lower tissue C:N ratios—a trait hypothesized to enhance microbial SOC decomposition. These interactions underscore the dual role of rhizosphere microbes in regulating N cycling and coupling it with soil carbon and nutrient dynamics.

#### 2.3.3. The Influence of Soil Phosphorus on Rhizosphere Microbes

Phosphorus (P) is an essential nutrient for plant and microbial life processes but frequently limits productivity in terrestrial ecosystems, particularly grasslands [39]. Soil phosphorus predominantly exists as sparingly soluble phosphates with low mobility, rendering it largely inaccessible for direct plant uptake. Soil microorganisms play a pivotal role in converting insoluble phosphates into plant-available forms, with plant-microbe symbioses being critical for P acquisition [40]. For instance, arbuscular mycorrhizal fungi (AMF) associated with plant roots expand the soil volume explored for phosphorus, enhance acid phosphatase activity, and elevate soil P availability, thereby facilitating rhizospheric P uptake [41]. Grazing can amplify phosphorus cycling rates by increasing phosphorus pools in plant seedlings and litter, enlarging soil P reservoirs at certain scales, and modifying abiotic-biotic interactions [42]. Plants enhance rhizospheric inorganic phosphate availability by releasing acid phosphatases, phytases, organic anions, or carboxylates, improving P nutrition for themselves and neighboring plants. Rhizospheric nutrient availability is closely tied to microbial activity—phosphate-solubilizing bacteria and AM fungi collaboratively mobilize P and boost its bioavailability [43]. In alpine meadow grasslands, grazing elevates rhizosphere soil nutrient availability [44]. While long-term grazing affects soil water retention and aggregate stability, its primary influence on microbial community composition manifests in rhizosphere soils. Grazing induces plants to allocate more carbon to the rhizosphere, stimulating extracellular enzyme activity and microbial metabolic rates, which drive soil P transformation and plant nutrient acquisition [45]. Metagenomic studies reveal that the abundance of microbial functional genes encoding phosphorus-cycling enzymes reflects the intensity of soil P-cycling processes. However, the mechanisms underlying grazing-mediated soil phosphorus transformation, particularly via rhizosphere microbial community modulation, remain poorly resolved.

#### 2.3.4. The Influence of Fertilization on Rhizosphere Microbes in Grassland Ecosystems

Root systems exhibit high phenotypic plasticity, enabling plants to adapt to dynamic environmental conditions, particularly soil nutrient availability and species interactions [46]. Fertilization is a critical strategy for enhancing productivity in grassland ecosystems. Over the past decade, increasing applications of nitrogen (N) and phosphorus (P) fertilizers have altered vegetation community structures and augmented the biomass of dominant species in grassland systems [47]. Nitrogen fertilization commonly reduces microbial biomass and bacterial diversity, likely mediated through soil acidification [48]. N amendment significantly restructures soil microbial community composition. Phospholipid fatty acid (PLFA) analyses reveal that N fertilization typically decreases fungal relative abundance, fungal-to-bacterial ratios, and/or the proportion of Gram-positive (G^+^) to Gram-negative (G^−^) bacteria (G^+^/G^−^ ratio) [49]. With increasing N inputs, the relative abundance of Proteobacteria increases while Acidobacteria declines [50]. According to microbial r-K strategy theory, bacteria are generally categorized as r-strategists, whereas fungi—characterized by slower growth rates—are often classified as K-strategists [51]. Similarly, G^+^ bacteria align with K-strategists, while G^−^ bacteria associate with r-strategists. Members of Proteobacteria and Acidobacteria are classified as representatives of copiotrophs and oligotrophs, corresponding to typical r- and K-strategists, respectively [52]. Nitrogen fertilization generally reduces the relative abundance of K-strategists and their ratio to r-strategists, while stimulating the dominance of fast-growing, copiotrophic r-strategists that thrive under nutrient enrichment.

Phosphorus fertilization influences rhizospheric phosphorus turnover and carbon-nitrogen coupling through modulation of microbial P-metabolism gene expression. Under low-P conditions, microorganisms upregulate phytase (*phoA*) and alkaline phosphatase (*phoD*) genes to mineralize organic P, while secreting carboxylates (e.g., citrate) to solubilize insoluble phosphates [52]. High-P environments suppress these genes but activate polyphosphate storage genes (e.g., *ppk*, encoding polyphosphate kinase) to promote intracellular polyphosphate accumulation [53]. Additionally, phosphorus availability shapes microbial energy allocation strategies by regulating C:N:P stoichiometric ratios. For instance, high-P conditions favor microbial biomass synthesis (r-strategy), enhancing extracellular polysaccharide secretion to compete for root-exuded carbon, whereas low-P conditions drive resource-acquisition adaptations (K-strategy), upregulating protease and cellulase activities to enhance organic matter decomposition [54]. Metabolomic analyses indicate that under P limitation, rhizosphere microbes produce strigolactone analogs to stimulate arbuscular mycorrhizal fungal (AMF) colonization, whereas high-P treatments elevate antibiotic synthesis (e.g., phenazines) to suppress pathogenic fungi [55].

## 3. The Influence of Biological Factors on Rhizosphere Microbes

### 3.1. The Influence of Plant Types on Rhizosphere Microorganisms

In grassland ecosystems, distinct plant functional types drive the directional assembly of rhizosphere microbial communities through divergent photosynthetic pathways, specialized root exudates, and signaling molecules, thereby modulating functional gene expression and microbial interaction networks [56,57]. C3 plants (e.g., *Lolium perenne*) secrete sucrose and phenolic acids at high concentrations, fostering dominance by Proteobacteria and Ascomycota fungi, which rapidly decompose labile carbon to accelerate soil organic carbon mineralization [58]. In contrast, C4 plants (e.g., *Miscanthus sinensis*) employ efficient CO_2_-concentrating mechanisms while exuding malate and succinate, promoting Actinobacteria and arbuscular mycorrhizal fungi (*Rhizophagus irregularis*) that stabilize recalcitrant carbon pools through mineral interactions [59]. Leguminous species (e.g., *Trifolium pratense*) recruit nitrogen-fixing symbionts via flavonoid-mediated signaling, enhancing ecosystem nitrogen input through microbial synergies [60]. Non-leguminous grasses secrete siderophores and oligosaccharides to establish beneficial consortia, fine-tuning rhizosphere C:N ratios while suppressing pathogen colonization [61].

Rhizosphere microbiomes exhibit marked spatial heterogeneity: root tip zones with elevated citrate secretion lower local pH by 0.5–1.2 units, enriching acidophilic taxa (Burkholderia) [62]. Plant-driven microbial modulation is climate-sensitive: under drought or +2 °C warming, C4 rhizospheres upregulate stress-resistance genes (proB, katE) and expand mycelial networks (30–50% denser) to enhance water acquisition. Chronic warming, however, disrupts C3 rhizosphere equilibrium (25% reduction in fungal/bacterial ratios), impairing ligninolytic enzyme activity and delaying carbon turnover [63]. Elevated CO_2_ (eCO_2_) stimulates C3 photosynthesis (12–18% increased rates), boosting exopolysaccharide-producing bacteria to enhance soil aggregation, while C4 rhizospheres partially compensate nitrogen limitation through diazotroph proliferation [64]. Thus, plant-mediated coevolution of metabolomes, microbiomes, and environments governs grassland carbon-nitrogen dynamics, nutrient efficiency, and stress resilience. However, long-term multi-omics integration (e.g., metagenomic-metabolomic linkages) is imperative to predict ecosystem stability under climatic shifts [9]. C3 and C4 plants face unique environmental pressures due to their different photosynthetic pathways. They enhance their adaptability to these stresses by recruiting specialized rhizosphere microbial communities: C4 plants (such as corn and sorghum) typically encounter high temperatures, drought, and nitrogen limitation. Their rhizosphere accumulates specific beneficial microorganisms. For instance, under nitrogen deficiency conditions, corn enriches the Massilia bacteria of the Oxalobacteraceae family through root exudation of flavonoids to promote lateral root development and nitrogen absorption [11].

### 3.2. The Influence of Plant Diversity and Community Structure on Rhizosphere Micrbes

Plant diversity and community structure drive rhizosphere microbial community composition and functional diversity. Higher plant diversity establishes diversified resource foundations for microbial communities through richer root exudate profiles. Root exudates (e.g., carbohydrates, organic acids, secondary metabolites, and signaling molecules) exhibit marked interspecific variations: leguminous plants release flavonoid compounds to specifically attract symbiotic nitrogen-fixers (e.g., *Rhizobium* genus), while grasses secrete phenolic acids that enrich cellulose-degrading Actinobacteria. Such resource heterogeneity induces microbial niche differentiation and functional complementarity, significantly enhancing rhizosphere microbial alpha diversity [65]. Diverse plant communities also reduce resource competition intensity among microbes, fostering mutualistic interactions (The core mechanism lies in the increase in resource heterogeneity and the formation of ecological niche differentiation). For instance, the symbiotic networks between arbuscular mycorrhizal fungi (AMF) and woody plants gain enhanced stability through diversified carbon inputs in mixed plantings [66]. However, this facilitative effect is modulated by the functional trait compatibility among plant species within the community: differences in rooting depth, C:N ratio, and litter decomposition rates further regulate microbial vertical stratification and metabolic preferences. Deep-rooted plants (e.g., *Quercus* spp.) provide refugia for drought-tolerant microbes by transporting deep-layer nutrients and water, whereas shallow-rooted grasses (e.g., *Lolium* spp.) stimulate phosphate-solubilizing bacteria (*Pseudomonas*) in topsoil through frequent exudation pulses [67].

The vertical stratification and species configuration of plant communities reshape rhizosphere microbial distribution and functionality through coupled physico-chemical-biological interactions. In tree-shrub-grass polyculture systems, root spatial segregation across strata creates heterogeneous microhabitats: canopy trees provide substrates for saprotrophic fungi via lignin-rich litter input, while herbaceous layers with dense fibrous roots significantly activate bacterial nitrogen-phosphorus transformation functions through intensive rhizodeposition [68]. Furthermore, plant interspecific interactions drive microbial community restructuring via allelochemical-mediated pathways: legume-grass intercropping enhances synergistic efficiency between diazotrophs (*nifH* gene carriers) and Glomeromycota fungi through nitrogen complementarity, whereas invasive plants (e.g., *Solidago canadensis*) suppress native rhizosphere symbionts (e.g., AMF) via allelopathic compounds, leading to microbial functional gene homogenization and ecosystem service deterioration [69].

Climate change factors (e.g., drought/warming) may destabilize established plant-microbe equilibria: deep-rooted plants maintain rhizodiversity through stable deep-water retention during droughts, yet intense precipitation events leach salt-tolerant microbes from shallow rhizospheres, significantly reducing their abundance [70]. Anthropogenic interventions (e.g., fertilization/monoculture) exacerbate these imbalances: excessive nitrogen input enriches oligotrophic Acidobacteria adapted to high-nutrient conditions but suppresses critical functional guilds (e.g., diazotrophs), ultimately diminishing soil nutrient cycling efficiency [71]. Current research urgently demands integration of multi-omics and in situ observation technologies to transcend spatiotemporal heterogeneity limitations. Combining metagenomics with metabolomics can decode regulatory pathways through which plant-specific exudates (e.g., strigolactones) control microbial gene expression, while rhizotron systems and X-ray CT imaging enable dynamic tracking of 3D root architecture evolution and microbial colonization patterns.

### 3.3. The Influence of Soil Fauna on Rhizosphere Microbes

Soil fauna reconfigure rhizosphere microenvironments through the physical perturbations induced by burrowing activities and exudate deposition, thereby modifying soil pore architecture, oxygen diffusion rates, and moisture gradients, which collectively govern microbial spatial distribution and functional dynamics. For instance, earthworm-derived stable macropores enhance oxygen accessibility and homogenize organic matter distribution, fostering the proliferation of aerobic bacteria (e.g., *Pseudomonas* and *Bacillus*) while suppressing the accumulation of anaerobic pathogens such as *Fusarium* [72]. Functioning as a “microbial bioreactor,” the earthworm gut selectively enriches and excretes specific microbial taxa during digestion, significantly augmenting the diversity of rhizosphere actinomycetes (e.g., *Streptomyces*) and lignin-decomposing fungi (e.g., Ascomycota), thereby accelerating organic matter mineralization and humification processes [73]. The activities of earthworms, especially the formation of earthworm castings, contribute to the expansion of arbuscular mycorrhizal fungi hyphal networks. At the same time, they influence the distribution and dissemination of arbuscular mycorrhizal fungi spores in the soil [74]. Through this symbiotic interaction network, plants obtain more abundant phosphorus nutrition. Healthier plants secrete more carbon sources through their root systems, which in turn provide more “fuel” for rhizosphere microorganisms (including bacteria and arbuscular mycorrhizal fungi), thereby consolidating and strengthening this mutually beneficial relationship [75]. Furthermore, collembolan grazing on fungal hyphae (e.g., ectomycorrhizal fungi) reshapes mycobial community composition, triggering plant roots to exude phenolic compounds that recruit antagonistic bacteria (e.g., *Burkholderia*) to compensate for disrupted mycorrhizal symbiosis, establishing ecological equilibrium through dynamic interspecies interactions [76]. Bioactive molecules secreted by soil fauna, including mucin proteins, digestive enzymes, and hormone analogs, serve as specialized carbon sources or signaling agents for rhizosphere microorganisms, modulating their metabolic pathways and functional gene expression. Earthworm-derived glycoproteins, for example, activate the bacterial *accA* gene (encoding acetyl-CoA carboxylase), enhancing lipid biosynthesis to fortify microbial membrane resistance against oxidative stress [77]. Nematode secretions such as ecdysteroids disrupt fungal G protein-coupled receptor (GPCR) signaling pathways, effectively suppressing hyphal proliferation of pathogenic fungi (e.g., *Rhizoctonia solani*) [78].

Conversely, microbial metabolites reciprocally regulate soil fauna behavior. Strigolactones released by arbuscular mycorrhizal fungi (AMF) attract collembolans to rhizosphere zones, where their grazing activities stimulate AMF hyphal network expansion, creating a microbial recruitment–faunal grazing–symbiotic synergy feedback loop that reinforces mutualistic benefits [79]. Predatory pressures exerted by soil fauna selectively modulate microbial community structure and functionality. For instance, bacterivorous nematodes (e.g., *Caenorhabditis elegans*) preferentially reduce the abundance of copiotrophic bacteria (e.g., Proteobacteria phylum) while promoting competitive proliferation of oligotrophic taxa (e.g., Acidobacteria phylum), optimizing carbon-to-nitrogen metabolic flux in the rhizosphere [80]. Moreover, soil fauna enhance the complexity and stability of microbial networks: mite-mediated dispersal of AMF spores and phosphate-solubilizing bacteria (PSB) facilitates plant-microbial symbiosis, whereas synergistic interactions between nematodes and earthworms inhibit biofilm formation by pathogens (e.g., *Ralstonia solanacearum*), thereby attenuating soil-borne disease incidence [81]. Notably, climate perturbations such as drought may alter soil fauna community composition, indirectly impairing their microbial regulatory capacity. For example, reduced earthworm activity under drought conditions diminishes the abundance of ACC deaminase-producing bacteria (e.g., *Pseudomonas putida*), which compromises plant drought tolerance by disrupting stress hormone modulation mechanisms [82].

### 3.4. The Influence of Grazing on Rhizosphere Microbes

Grazing represents one of the fundamental utilization strategies for grassland ecosystems. Livestock alter plant diversity, soil nutrient cycling, and microbial community composition through foraging, trampling, and fecal deposition [83]. Grazing modifies carbon and nitrogen cycling patterns at specific spatial scales, enhancing nutrient turnover through biotic-abiotic interactions [84]. Grazing intensity exerts significant effects on rhizosphere bacterial diversity: bacterial abundance in rhizospheric soils increases with grazing pressure, whereas no significant changes occur in bulk soil bacteria or fungi. Under heavy grazing regimes, the abundance of total microbial communities, bacteria, and fungi declines substantially. Long-term intensive grazing drastically reduces plant biomass, as herbivores damage aboveground plant organs, leading to diminished allocation of assimilated carbon to belowground tissues. This carbon limitation reduces substrate availability for rhizosphere bacteria, ultimately decreasing bacterial diversity [9]. Notably, plant diversity typically correlates positively with microbial diversity at the community level. While moderate disturbances theoretically enhance biodiversity under the Intermediate Disturbance Hypothesis, chronic grazing—even at low intensities—markedly reduces species diversity in desert steppes. Grazing-induced depletion of plant diversity diminishes carbon source heterogeneity for bacterial taxa [85], causing competitive exclusion of specialized bacterial groups and subsequent rhizobacterial diversity loss. Fungal communities demonstrate higher grazing tolerance (or lower sensitivity) compared to bacteria, with grazing promoting fungal proliferation, particularly in rhizospheric soils. Given their inherently low biomass and vegetation cover, desert grasslands exhibit accentuated responses to grazing, manifested as significant divergence in diversity and composition between rhizosphere and bulk soil microbial communities [86]. Excessive grazing can cause the soil microbial community to shift from being dominated by fungal food webs to being dominated by bacterial food webs. In particular, arbuscular mycorrhizal fungi are crucial for the formation of soil aggregates and the decomposition of stable carbon. The decline in the proportion of fungi will weaken the soil’s carbon sequestration capacity and structural stability. The relative abundance of the Actinobacteria phylum in the soil decreases, while the abundance of the Deinococcus and Firmicutes phyla increases. Actinobacteria are usually associated with the decomposition of complex organic matter, and their reduction may indicate the decline of soil nutrient cycling functions [87]. Additionally, in studies of alpine meadows on the Qinghai–Tibet Plateau, it was found that the number of Flavobacteriaceae in moderately and severely grazed areas was significantly higher than in lightly and extremely heavily grazed areas [88]. Rhizosphere microbial diversity indices, including richness and Shannon diversity, are consistently lower than those in bulk soils. This pattern suggests that microbial diversity diminishes with proximity to plant roots, potentially because the rhizosphere microbiome is selectively filtered from bulk soil communities under host plant regulation.

## 4. Prospects

**Figure 1:**
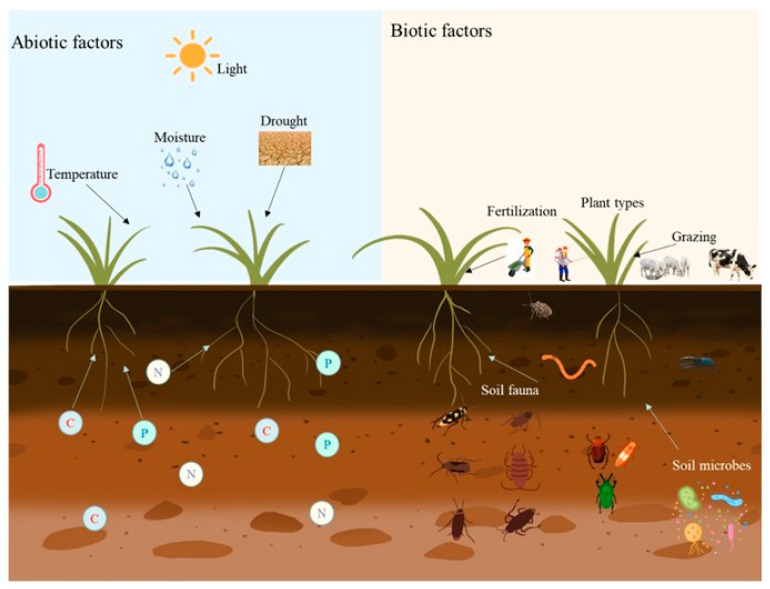
The influence of biological and abiotic factors on grassland ecosystems.

Under the combined pressures of climate change and anthropogenic activities, intensified interactions between biotic and abiotic factors amplify the complexity of rhizosphere microbial responses (Figure 1). For instance, synergistic effects of nitrogen deposition and elevated CO_2_ concentrations enhance the belowground allocation of plant photoassimilates (e.g., increased root exudation), transiently stimulating microbial activity. However, chronic nitrogen enrichment induces soil acidification and microbial carbon-nitrogen metabolic dysregulation (e.g., reduced C/N enzyme activity ratios), ultimately leading to diminished functional redundancy. Furthermore, agricultural management practices (e.g., crop rotation, tillage) and land-use changes (e.g., grassland-to-cropland conversion) disrupt rhizosphere continuity, altering microbial source-sink dynamics and compromising hyphal network connectivity and system resilience. Future research should prioritize the integration of multi-omics data and ecosystem models to elucidate adaptive evolutionary trajectories of rhizosphere microbiomes under multifactorial stresses, and to advance microbiome-based restoration frameworks for degraded grasslands.

## Data Availability

No new data were created or analyzed in this study. Data sharing is not applicable to this article.
